# Supplementary material for: Does the Chimerization Process Affect the Immunochemical Properties of WNV-Neutralizing Antibody 900?
Source: Int J Mol Sci. 2025 Dec 18;26(24):12181. doi: 10.3390/ijms262412181 (PMC12733864; doi:10.3390/ijms262412181)
Supplement: Supplementary file 1 [file ijms-26-12181-s001.zip › Supplementary material S2. SEC.pdf]

Supplementary material

a

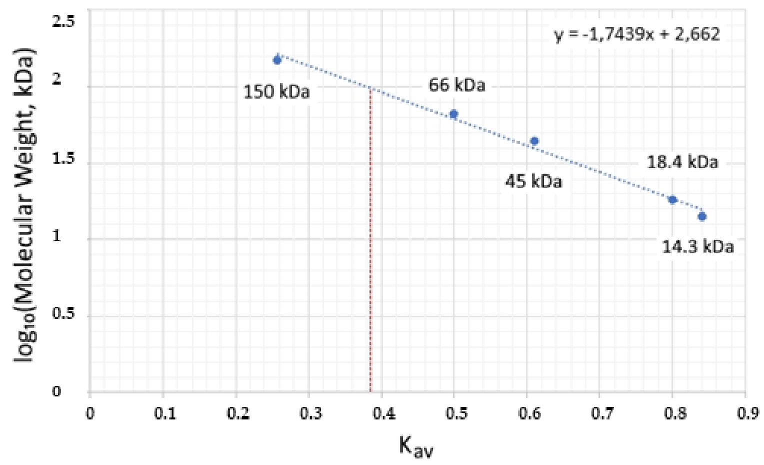

b

Standart	Molecular weight, kDa	Ve, mL
ɣ- globulin	150	1.01
BSA	66	1.63
Egg albumin	45	2.02
β-lactoglobulin	18.4	2.84
lysozyme	14.3	3.17
**Void Volume (V0)**		0.85

c

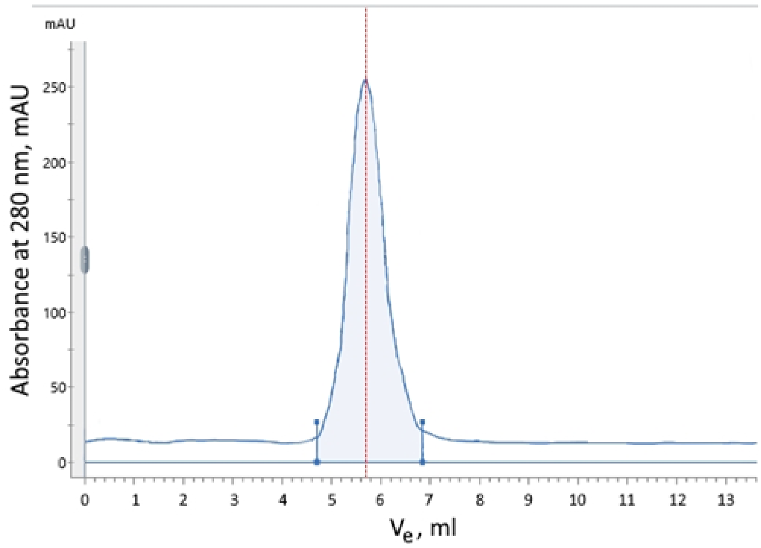

d

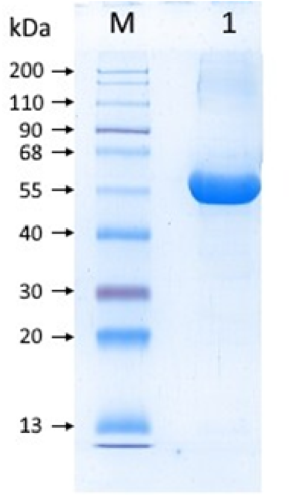

Figure S2. Size-exclusion chromatography of purified mAb 900. (a) SEC Calibration Curve; (b) Standards for constructing the calibration curve; (c) SEC chromatogram of mAb 900; (d) SDS-PAGE of mAb 900 in denaturing conditions. Lane M - Protein Marker, 10 to 200 kDa, lane 1 - mAb 900.
