# Supplementary material for: Does the Chimerization Process Affect the Immunochemical Properties of WNV-Neutralizing Antibody 900?
Source: Int J Mol Sci. 2025 Dec 18;26(24):12181. doi: 10.3390/ijms262412181 (PMC12733864; doi:10.3390/ijms262412181)
Supplement: Supplementary file 1 [file ijms-26-12181-s001.zip › Supplementary material S3. CD Tm.pdf]

Supplementary material

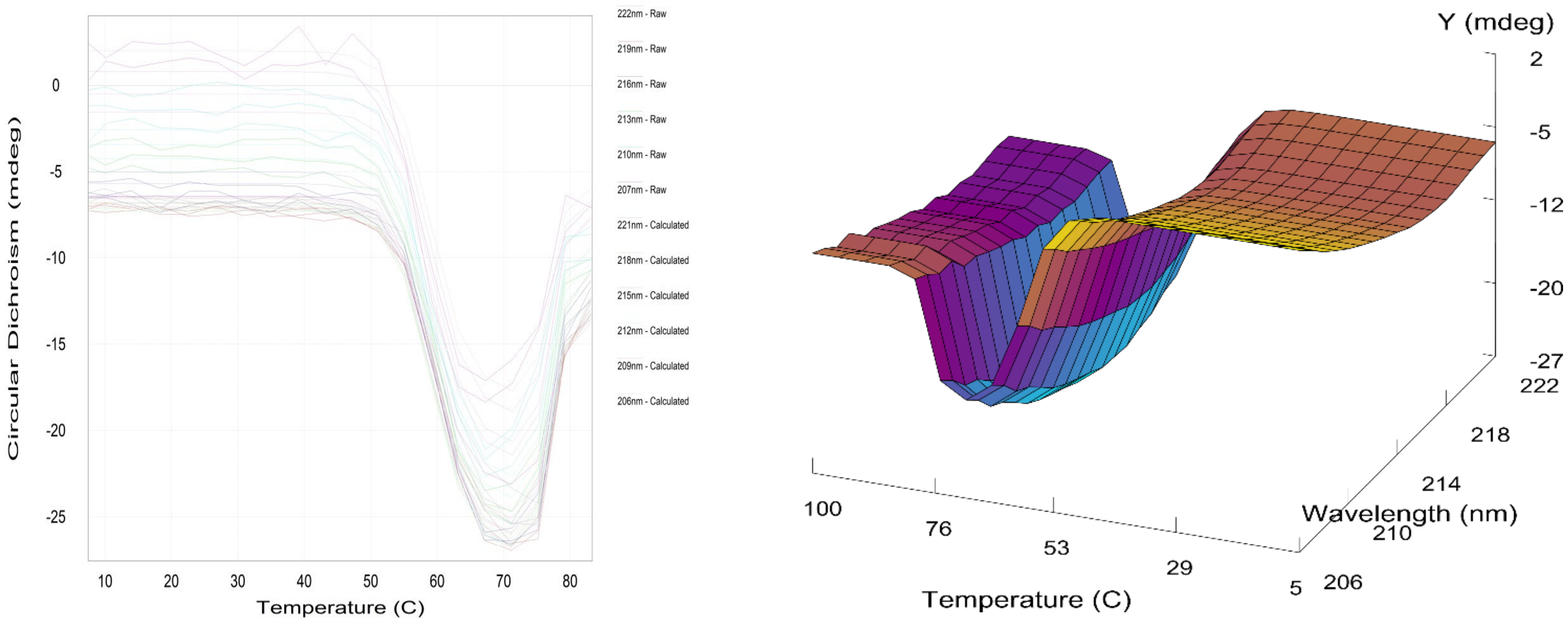

Figure S3. Analysis of the dependence of CD spectra on temperature using the Global3 program.
