# Supplementary material for: Does the Chimerization Process Affect the Immunochemical Properties of WNV-Neutralizing Antibody 900?
Source: Int J Mol Sci. 2025 Dec 18;26(24):12181. doi: 10.3390/ijms262412181 (PMC12733864; doi:10.3390/ijms262412181)
Supplement: Supplementary file 1 [file ijms-26-12181-s001.zip › Supplementary material S4. Glycan profile of mAb 900.pdf]

Supplementary material

Table S1. Content of non-fucosylated and galactosylated glycans in mAb 900 samples.

	mAb 900, sample 1	mAb 900, sample 2
**Glycan**	**Relative glycan content, %**	**Relative glycan content, %**
G0-N	0.2	0.2
G0F-N	0.5	0.7
G0	4.5	4.3
G0F	58.5	50.4
Man5	0.9	0.5
G1	1.6	1.8
G1F	27.0	32.5
G2F	4.5	6.5
afucosylated glycans content (AF, %)	7.1	6.9
galactosylated glycans content (G, %)	37.6	47.3

Results of determination of the glycan profile of mAb 900 for N-glycans

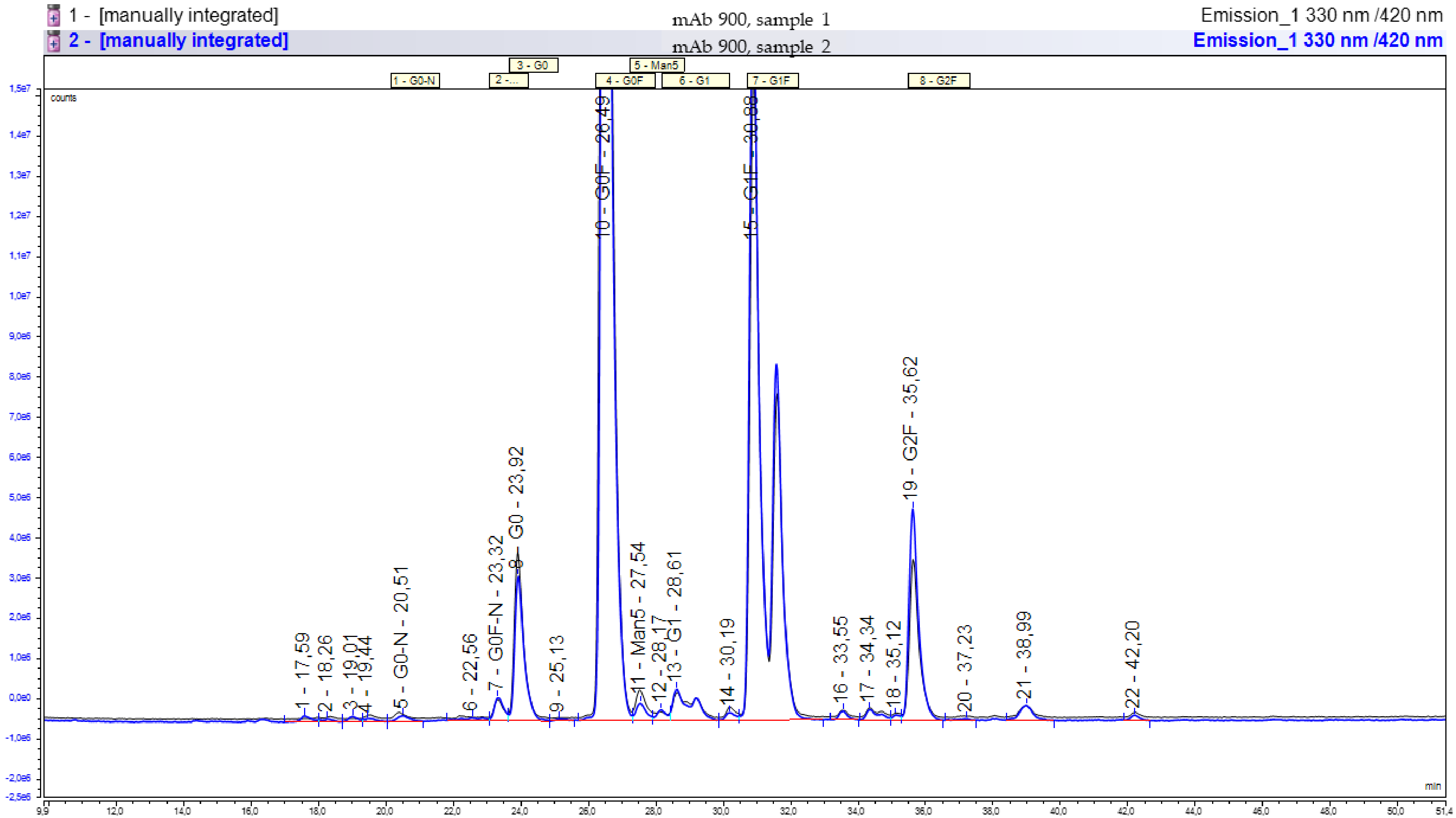

Figure S4. Overlay of chromatograms of the N-glycan profile of mAb 900 sample 1 and mAb 900 sample 2.
