# Supplementary figures and images for: Does the Chimerization Process Affect the Immunochemical Properties of WNV-Neutralizing Antibody 900?
Source: Int J Mol Sci. 2025 Dec 18;26(24):12181. doi: 10.3390/ijms262412181 (PMC12733864; doi:10.3390/ijms262412181)

Supplementary material

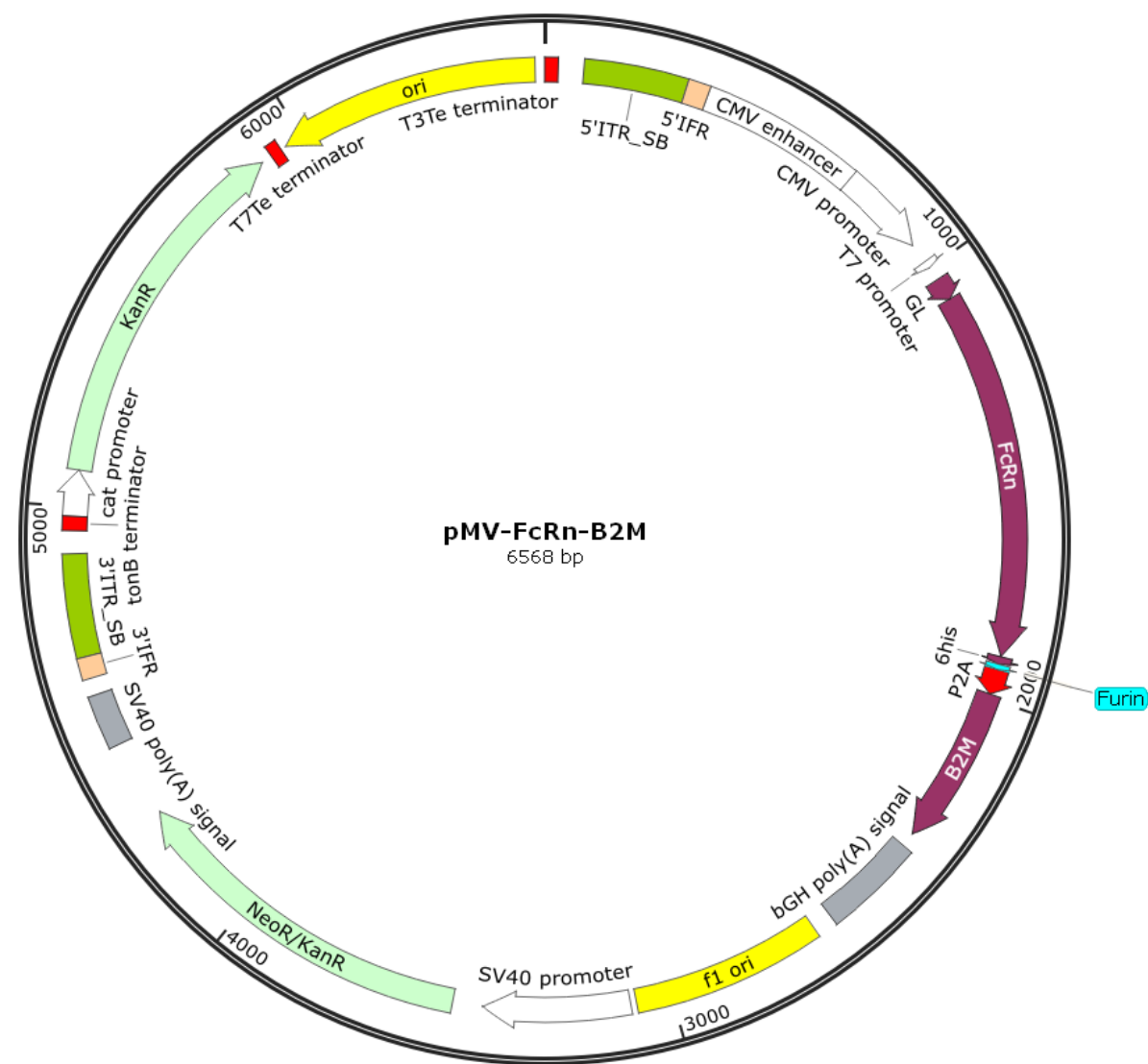

Figure S1. Map of integration vector pMV-FcRn-B2.

Supplement: Supplementary file 1 [file ijms-26-12181-s001.zip › Supplementary material S1. Map of integration vector pMV-FcRn-B2M.pdf]
